# Word Structure Tunes Electrophysiological and Hemodynamic Responses in the Frontal Cortex

**DOI:** 10.3390/bioengineering10030288

**Published:** 2023-02-23

**Authors:** Fei Gao, Lin Hua, Yuwen He, Jie Xu, Defeng Li, Juan Zhang, Zhen Yuan

**Affiliations:** 1 Centre for Cognitive and Brain Sciences, University of Macau, Macau SAR 999078, China; 2 Institute of Modern Languages and Linguistics, Fudan University, Shanghai 200433, China; 3 Faculty of Health Sciences, University of Macau, Macau SAR 999078, China; 4 Faculty of Arts and Humanities, University of Macau, Macau SAR 999078, China; 5 Faculty of Education, University of Macau, Macau SAR 999078, China

**Keywords:** morphological priming, word structure, derivation, compound, EEG-fNIRS

## Abstract

To date, it is still unclear how word structure might impact lexical processing in the brain for languages with an impoverished system of grammatical morphology such as Chinese. In this study, concurrent electroencephalogram (EEG) and functional near-infrared spectroscopy (fNIRS) recordings were performed to inspect the temporal and spatial brain activities that are related to Chinese word structure (compound vs. derivation vs. non-morphological) effects. A masked priming paradigm was utilized on three lexical conditions (compound constitute priming, derivation constitute priming, and non-morphological priming) to tap Chinese native speakers’ structural sensitivity to differing word structures. The compound vs. derivation structure effect was revealed by the behavioral data as well as the temporal and spatial brain activation patterns. In the masked priming task, Chinese derivations exhibited significantly enhanced brain activation in the frontal cortex and involved broader brain networks as compared with lexicalized compounds. The results were interpreted by the differing connection patterns between constitute morphemes within a given word structure from a spreading activation perspective. More importantly, we demonstrated that the Chinese word structure effect showed a distinct brain activation pattern from that of the dual-route mechanism in alphabetic languages. Therefore, this work paved a new avenue for comprehensively understanding the underlying cognitive neural mechanisms associated with Chinese derivations and coordinate compounds.

## 1. Introduction

Morphology constitutes an important component of the human language system, which concerns not only how words are formed but also how they are inter-connected with each other in the arguable mental lexicon [1,2,3]. A key issue pertaining to morphological processing in the past five decades has discussed whether morphologically complex words are stored in a holistic or decomposed manner [4,5], which has yet to reach consensus [6,7]. Importantly, irrespective of whether decomposition is an obligatory process of word recognition, the way how morphemes/lexemes are connected to establish a new word has been recognized to significantly modulate lexical access in the human mind/brain [8,9]. However, it is still unclear how different morphological structures might impact lexical processing in the brain for languages with an impoverished system of grammatical morphology such as Chinese. Therefore, this study set out to explore the neural underpinnings of morphological structure processing in Chinese words.

To shed light on the sub-types of word morphological structures, behavioral and neuroimaging studies have been carried out to inspect the representations of inflectional and derivational words in alphabetical languages [8,10,11,12,13]. Inflections are used upon word forms to mark grammatical changes (e.g., gender, number, voice, and tense), while derivations are characterized by attaching affixes (prefixes, infixes, and suffixes) to base words, thus establishing a new word form, meaning, and even grammatical category. For example, “*walks*” in “*Tom walks to school every day*” is an inflection marking a third-person singular verb in the present tense, while “*walker*” is a noun derived from the verb “*walk*”. Specifically, Newman et al. [14] compared the event-related potential (ERP) difference between the regular/irregular past tenses of English verbs, phrase structure, and lexical semantics in sentential contexts. It was found that irregular past-tense word forms elicited significant left-lateralized anterior negativities (LANs) on the human scalp, probably indexing a rule-based computation on morphology, while significant P600 effects identified from regular and irregular violations might suggest a controlled processing for lexicalized linguistic items (e.g., irregular forms such as *ran*). This pattern was replicated in a recent Swedish study [15] and elaborated in a so-called dual-access model [16,17], highlighting the neurocognitive signatures for morphological complexity. In this dual system, the processing of regular inflections in English might selectively engage the left fronto-temporal network, which is sensitive to rule-based decomposition and combination. By contrast, the bilateral subsystem and broader brain regions are employed in reading derivational words and highly lexicalized forms, which are neurobiologically specified for whole-word and storage-based sound-to-meaning mapping.

As compared with research on inflections and derivations, fewer studies have been performed to examine the neural correlates of compound word processing and differentiate the sub-types of compounding structure. Compounding involves the process of concatenating different lexical constituents (words/lexemes) to create new lexical items, which might represent a fundamental mechanism of morphological productivity for most languages [18,19]. The majority of existing studies focused on how constitute frequency [20], semantic transparency [21,22,23], and constitute headedness [21,24] would impact lexical access and morphological parsing in compound word recognition [8]. Now what remains unclear is the extent to which word structure (i.e., the grammatical and semantic relations between lexical constituents) would modulate the representation and processing of compound words. Insights from conventional descriptive linguistics [25] suggested a variety of compound structures, partially based on syntactic relations yet mostly on semantics, including apposition (e.g., *woman doctor*), subject/object + action (e.g., *sunrise*), purpose (e.g., *wineglass*), and others. However, existing psycholinguistic studies on compound processing exclusively focused on the “modifier-head” (e.g., *teacup*, *pineapple*) structure [22,23]. Although a couple of studies mentioned the existence of coordinate structure in English, or the so-called “dual compounds”/“copulative type” (e.g., *singer-songwriter*, *architect-sculptor, in-and-out*) that is primarily a hyphenated combination [3,18,25], no study has been conducted to inspect the neural basis of this structure or make comparisons between various compounding structures. The inadequate evidence on this issue might be attributable to the relatively impoverished compounding system of English words.

More interestingly, the Chinese language is categorized as one without much inflection, which is in striking contrast to alphabetical languages. According to structuralism linguistics [26], Chinese vocabulary is classified into four categories in terms of word structure, including mono-morphemic words (e.g., 葡萄, pu2-tao, “grape”), derivation (prefixed and suffixed), reduplication (e.g., 妈妈, ma1-ma, mom-mom, “mother”), and compound, among which compound words constitute the largest portion (more than 70%). According to a survey throughout the modern Chinese dictionary [27], the majority of multi-morphemic words in the colloquial dataset are compound words (83.02%), followed by derivations (14.51%). In particular, there are five sub-structures of Chinese compounds, including subordination (54%, e.g., 黑板, hei1-ban3, black-board, “black-board”), coordination (26%, e.g., 花草, hua1-cao3, flower-grass, “plant”), verb-object (18%, e.g., 吃饭, chi1-fan4, eat-food, “to eat”), verb-resultative (2%, e.g., 长大, zhang3-da4, grow-up, “grow up”), and subject-predicate (1%, e.g., 晚安, wan3-an1, night-safe, “good night”) statistics from [28]. The seminal work concerning Chinese word structure effects on morphological decomposition revealed that compounding structure (coordination vs. subordination) might modulate the constitute frequency effect in word recognition [29]. Specifically, it was discovered that the frequency of both constituents of a coordinate structure impacted the reaction time (RT) to target words, whereas in the subordinate structure, RT was only related to the constitute frequency in the final position. These findings shed light on the decomposition mechanism of Chinese compound words, which is also mediated by morphological structures. More importantly, both behavioral and neuroimaging studies since then have accumulated ample evidence for the cognitive neural mechanism associated with the morphemic effect of Chinese word reading. For example, ambiguity and polysemantic properties at the constitute morpheme level would significantly affect the representation of disyllabic compound words [30,31,32,33,34,35,36,37]. In addition to the morphemic effect, morpheme relations were also investigated in terms of semantic/thematic [38,39,40,41,42] and grammatical aspects.

In addition, existing behavioral and electrophysiological studies have mostly concentrated on subordinate and coordinate compounding structures and their corresponding representational differences in the mental lexicon. Drawing on a compounding production task, Liu and McBride-Chang [43] compared Chinese third graders’ word production performance with four compounding structures. Their results revealed that subordinate and coordinate structures were easier to produce than subject–predicate and verb–object structures, whose difficulty was detected to be proportional to their distributional characteristics in language units [44]. Subsequent studies differentiated the subordinate and coordinate structures by using various paradigms. For example, subordinate structure was harder to recognize than coordination in a priming lexical decision task where semantic relatedness and structural consistency between primes and targets were manipulated [45]. In the subordinate condition, the same structures facilitated the semantic priming effect while the coordinate structures manifested the opposite pattern. Meanwhile, subordinate structures significantly boost literacy performance in memorizing compound words [46,47], relative to coordinate structures. Liu [46] attributed this difference to the strengths of constituent connections within a compound word in light of the spreading activation theory [48] and further proposed the independent representation of morphological structure in the mental lexicon [47]. Two constituent morphemes in a coordinate structure contribute equally to the whole word meaning (e.g., 风雨, feng1yu3, wind-rain, “storm”), whose inter-connections are relatively weaker than the modifier-head relation in subordinate structure. It therefore requires more cognitive efforts to combine the lexical constituents for coordinate structures. Importantly, there might exist a morphological structure layer in the mental lexicon [47], in addition to the morpheme and word layers, which were originally proposed by the interactive activation model [49]. At this specific layer for word structures, both the semantic and syntactic connections between lexical constituents would be activated. Given the relatively weaker semantic connections in coordinate structure relative to subordinate structure, its corresponding morphological effect might be dampened.

However, the different patterns between coordinate and subordinate structures from Liu et al. were not replicated by Cui et al. [50] and Chung et al. [3]. The inconsistency was thought to result from the prolonged stimulus onset asynchrony (SOA = 200 ms). For Chung et al.’s ERP study at a short SOA (57 ms), only coordinate compounds were used, and the morphological structure effect was null in behavioral data although manifested by the P250 effect (220–300 ms time windows). Structural priming elicited greater P250 than distinct structure pairs, which might indicate word structure facilitation and top-down processing at the early stage of lexical access [51]. The discrepancy calls for further examination concerning the morphological structure effect in coordinate compounds.

Only a handful of neuroimaging studies have quantified the brain changes associated with Chinese morphological processing [36,52,53,54]. They identified that both the left frontal and temporal cortex are involved in processing Chinese morphology, yet failed to differentiate various structures (i.e., mixed with different sub-structures of compounding and derivations). To our knowledge, there is only one functional neuroimaging study that tried to disassociate the brain responses to various sub-structures of Chinese compound words [2]. Hsu and colleagues examined the Chinese morphological complexity effect (mono-morphemic vs. multi-morphemic) and compounding structural effect (subordinate, coordinate, and verb-object) by using a lexical decision task and magnetoencephalography (MEG) technique. It was found that compound words generated greater brain activations at an early stage (200 ms) in the left temporal cortex. In the later time window of 300–400 ms, however, there was a null effect in coordinate structure as compared with that from the baseline (mono-morphemic word), while both subordinate and verb-object structures generated greater responses in the posterior part of the left temporal region. This work attributed the absence of the coordinate structure effect in the left temporal cortex to the fact that coordinate compound words might lack a specifier-head-complement relational structure as manifested in subordinate and verb-object words. Instead, the two component morphemes make equal contributions to the whole-word meaning, resulting in a rather loose connection. However, this study failed to include the frontal cortex as a region of interest (ROI) in brain activation analysis, while related studies revealed that the left frontal cortex is also a crucial hub for Chinese morphological processing [52,54], suggesting that it is essential to examine the role of the frontal cortex in processing different compound sub-types.

Therefore, the neurobiological basis of differing Chinese morphological structures is still poorly understood with respect to both temporal signatures and localizations in the human brain. Among different compounding structures, much uncertainty still exists regarding the neural reality of the coordinate structure effect. Additionally, no previous study has investigated the brain representations of Chinese derivational words, even though they constitute the second largest proportion of Chinese vocabulary. The present study therefore aims at exploring whether Chinese derivations employ the same cognitive resources as compounds with respect to the inter-connections of their constituent morphemes. Specifically, only coordinate structures were selected for the compounding condition, while suffixed structures were chosen for the derivational condition. Masked priming was used to elicit early and automatic morphological parsing [55,56,57] in a visual lexical decision task, which was able to tap the participants’ structural sensitivity in an unconscious mode. Meanwhile, we manipulated the prime-target relationship across three conditions: constitute priming in derivational words, constitute priming in coordinate-compounds, and a non-morphological relationship (control). As primes and targets were semantically related across the three conditions, the only difference between the conditions would be the morphological relationship. Electroencephalogram (EEG) and functional near-infrared spectroscopy (fNIRS) were recorded simultaneously to depict both temporal signatures and spatial activations in the brain, as the latter would compensate for the relatively poor spatial resolution of EEG by virtue of the neurovascular coupling mechanism [58]. As such, we wished to inspect the temporal and spatial specifications in the human brain associated with the effect of Chinese word structure (derivation vs. compound vs. non-morphological). Importantly, research on Chinese morphology, because of its high contrast with alphabetic systems, could support or qualify conclusions based on alphabetic languages [59,60,61]. Our findings would advance our understanding of the neural signature of Chinese word reading and language comprehension, which further informs the universal science of reading to a great extent.

## 2. Materials and Methods

### 2.1. Participants

Thirty Mandarin Chinese native speakers (mean age: 22.2 ± 3.2 years old; 15 females) were recruited from the University of Macau campus. They were registered university students from various majors. All participants were right-handed and reported no neurological illness or mental disorder, with normal or corrected-to-normal vision. All materials and procedures were approved by the Institutional Review Board at the University of Macau. A written consent form was obtained from each participant prior to the experiment.

### 2.2. Stimuli Materials

First, 36 disyllabic Chinese coordinate-compound words and 36 disyllabic derivational words with suffixes (e.g., 儿/er/, 民/min2/) were selected. In a masked priming task [62], these words were primed by their corresponding constituent morpheme in the head position. In addition, 36 disyllabic words were selected as the control condition, with a semantically related Chinese character as the prime. In the control condition, the prime character and the constituent of the target word were distinct at both orthographic and phonological levels and yielded no morphological relationship. Twelve native Chinese speakers were invited to rate the semantic relatedness between the prime and target among 36 derivations, 36 coordinate compounds, and 36 control items on a 0–7 Likert scale. The averaged semantic relatedness for each condition was above five, indicating high semantic transparency and consistency across conditions. In particular, the three conditions were matched regarding word frequency, character frequency, and number of strokes (Table 1).

Besides, 108 disyllabic Chinese non-words were selected from MELD-SCH dataset [63]. The non-words yielded pronounceable meaningless strings and would be primed by a distinct character. They worked as fillers to balance the yes/no responses.

### 2.3. Procedures

**Figure 1:**
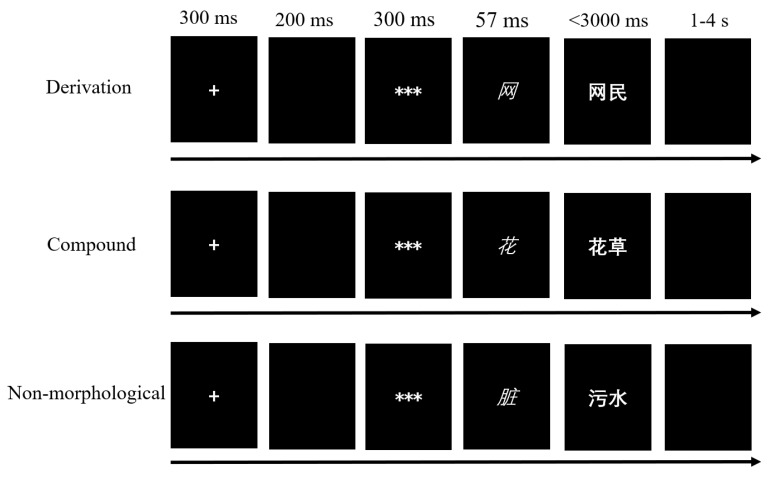
Schematic of the lexical decision task. Participants need to decide whether the target is a real word or not when they read the character string. The stimulus-onset asynchrony (SOA) between prime and target is set at 57 ms in light of the previous study [3].

The visual lexical decision task was adapted from the morphological priming paradigm with a short SOA [3] and a masked priming technique [62]. E-prime was used to program the materials and procedures (Figure 1), in which a white fixation was first presented in the screen center of a PC for 300 ms, followed by a blank of 200 ms. And then a series of asterisks serving as the mask were presented for 300 ms, and subsequently, the mask would be replaced by the prime lasting for 57 ms. After the prime disappeared, participants would read the target and decide whether the displayed character string is a real Chinese word or not by pressing the corresponding buttons labelled as “yes” or “no” in the keyboard as quickly as possible. If they failed to react within three seconds, it would be marked as a wrong response automatically by the task. After the response, there would be blank jittered from one to four seconds [64]. All primes were presented with the italic *Kaiti* typeface and a size of 40, whereas targets were displayed with the bold *SimHei* at 40. Ten practice trials with correctness feedback were offered before the formal experimental tests.

### 2.4. EEG Recordings and Data Analysis

**Figure 2:**
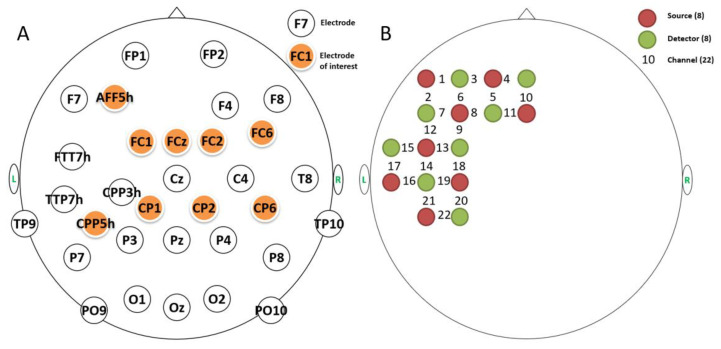
The layout of fused EEG-fNIRS arrangement. (**A**) There were 32 electrodes in total, nine of which (in orange) were used for further ERP analysis. (**B**) Eight light sources and 8 detectors generated 22 fNIRS channels, covering the frontal and temporal regions of the left hemisphere.

EEG and fNIRS data were collected simultaneously using an EasyCap (Brain Products, Munich, Germany), which was connected to the Brain Products EEG device and NIRScout system (NIRx Medizintechinik GmbH, Berlin, Germany). For EEG acquisition, 32 electrodes were arranged on the cap based on the international 10/20 system (Figure 2A). EEG data were digitized at 500 Hz with a bandpass filter of 0.03–70 Hz. During the online acquisition, the left mastoid electrode was used as a reference, and the impedance of all electrodes was kept below 20 kΩ.

For offline analysis in EEGLAB v2021.0, continuous EEG data were first re-referenced to the average of all channels and filtered with a band pass of 1–30 Hz. Bad channels were interpolated by averaging the spherical electrodes, which took up less than 3% of all channels. Then data segmentation was completed, which consisted of 158 ms before the target onset and 1000 ms afterwards. Eye movement components were removed from the segmented data by the Independent Component Analysis (ICA) algorithm and ICLabel plugin [65]. Bad epochs were further removed by visual inspection.

The averaged amplitudes of selected electrodes were computed and compared in the time windows of 220–300 ms (P250) and 300–500 ms (N400) according to detected ERP components [3]. Specifically, AFF5h, FC1, FCz, FC2, and FC6 from the bilateral and midline sites of the frontal cortex were examined for the ERP component P250, while centro-parietal electrodes CPP5h, CP1, CP2, and CP6 were inspected for the N400 effect.

### 2.5. fNIRS Recording and Data Analysis

Eight LED light sources and eight detectors were placed in the left frontal and temporal cortex [64] to cover the classic semantic and morphological networks [52,53], thus generating 22 fNIRS channels (Figure 2B). Each source transmitted LED lights at the wavelengths of 760 nm and 850 nm. The distance between each light source and detector was 3 cm. Optical signals were acquired at a sampling rate of 7.81 Hz. The MNI coordinates of all optodes and channels were obtained from their spatial information at the international 10/20 system, which were then imported into the NIRS_SPM software [66] to generate anatomical labels and percentages of overlap.

The pre-processing of fNIRS data was performed with nirsLAB [67]. Data from one participant was excluded due to extensive physiology noise, leaving a dataset of 29 participants for further analysis. Each fNIRS segment lasted for 14 s, consisting of 1s before prime onset and 13 s for the hemodynamic response period afterwards. Motion artifacts were removed from the raw data by the built-in algorithm and were subsequently filtered with a band pass of 0.01–0.2 Hz. Oxygenated hemoglobin (HbO) and deoxygenated hemoglobin concentration (HbR) changes were modeled in the Level 1 module of statistical parametric mapping with the canonical HRF function. As a result, general linear model (GLM) coefficients (beta values) were obtained across all priming conditions from each participant.

## 3. Results

### 3.1. Behavioral Results

**Figure 3:**
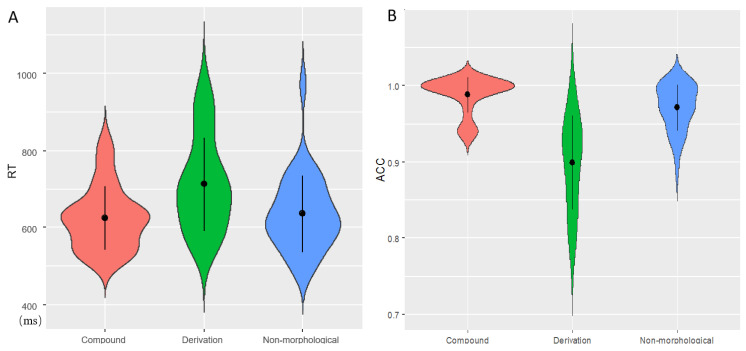
Violin plots of behavioral results. Black points denote means, whereas the error bars represent standard deviations. RT (**A**) and ACC (**B**) were visualized across three conditions.

The grand-average accuracy rate (ACC) was 95.26%, indicating that participants were well engaged in the task. One-way repeated-measures analyses of variance (ANOVA) with lexical conditions (compound constitute priming, derivation constitute priming, and non-morphological priming) were conducted on RT and ACC, respectively. A significant main effect of lexical condition on RT was detected, *F*(2, 58) = 34.085, *p* < 0.001, partial *η*^2^ = 0.540. Pairwise comparisons (Figure 3A) revealed that RT to derivational priming (712 ± 121 ms) was significantly longer than compound constitute priming (625 ± 82 ms) and non-morphological priming (636 ± 99 ms) (*p*s < 0.001), whereas there was no significant difference between compound and non-morphological priming (*p* > 0.05).

Likewise, the lexical condition effect was also identified for ACC, *F*(2, 58) = 37.873, *p* < 0.01, partial *η*^2^ = 0.566. Derivational priming (89.87 ± 6.19%) exhibited significantly lower ACC as compared with compound (98.8 ± 2.31%) and non-morphological (97.1 ± 3.03%) cases (*p*s < 0.001). Meanwhile, ACC of non-morphological priming was significantly lower than that of the compound case (*p* < 0.01). The behavioral results were visualized in Figure 3.

### 3.2. ERP Results

The P250 effect was inspected during the time window of 220–300 ms after target word onset in both bilateral and midline sites of the frontal region [3]. A two-way repeated-measures ANOVA was performed with lexical condition (compound constitute priming, derivation constitute priming, and non-morphological priming) and hemisphere at the bilateral sites (left: AFF5h, FC1; right: FC2, FC6) as independent variables. Although no significant lexical condition effect was revealed [*F*(2, 58) = 2.169, *p* = 0.131, partial *η*^2^ = 0.07], the main effect of hemisphere was identified, *F*(1, 29) = 0.252, *p* <0.05, partial *η*^2^ = 0.153. In particular, the right hemisphere (2.55 ± 0.31 µV) showed a larger P250 than the left one (1.92 ± 0.30 µV). Meanwhile, significant interaction between lexical condition and hemisphere was detected, *F*(2, 58) = 4.642, *p* = 0.015, partial *η*^2^ = 0.138, such that the non-morphological condition (2.10 ± 0.33 µV) elicited significantly higher P250 than the compound case (1.54 ± 0.37 µV) over the left hemisphere, while derivational priming (2.97 ± 0.37 µV) evoked significantly greater P250 than the compound case (2.27 ± 0.34 µV) along the right hemisphere. In contrast, no other comparisons were significant. Likewise, the effect of lexical condition was examined at the midline electrode (FCz) of the frontal cortex, detecting no reliable difference, *F*(2, 58) = 1.262, *p* = 0.289, partial *η*^2^ = 0.042.

**Figure 4:**
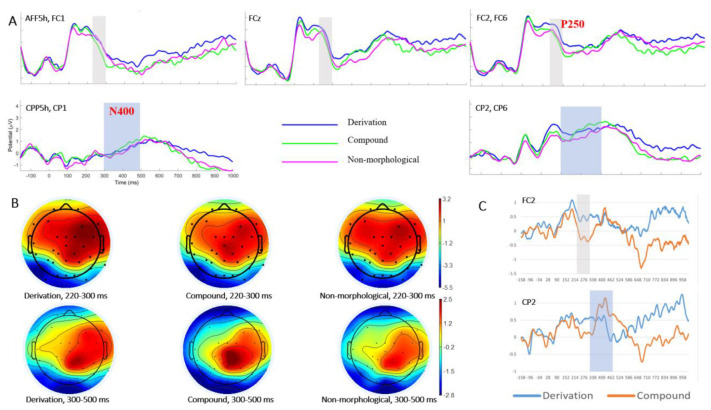
Visualization of ERP results. (**A**) Grand averaged ERPs over the frontal electrodes (the first row) demonstrating P250 effect (in grew shades), and centro-parietal sites (the second row) manifesting N400 activities (in blue shades). (**B**) Brain topographies of P250 (the first row) and N400 (the second row) across three conditions. (**C**) Difference waves for Derivation vs. Control (in blue), and Compound vs. Control (in orange) at electrodes FC2 and CP2, respectively. The time windows of P250 and N400 were shaded in grey and blue, respectively.

Regarding grand-averaged ERP and topographies (Figure 4A,B) as well as previous studies [3], N400 effect was examined in the time window of 300–500 ms with lexical condition and hemisphere (left: CPP5h, CP1; right: CP2, CP6) as factors. There was a significant hemisphere effect, *F*(1, 29) = 40.433, *p* < 0.001, partial *η*^2^ = 0.582, such that lower N400 was detected in the left hemisphere (0.295 ± 0.268 µV) than that in the right one (1.756 ± 0.278 µV). No other significant main effect or interaction was detected.

Furthermore, the priming effects of derivational and coordinate-compound structures were assessed by quantifying the difference in ERP waves (P250 and N400) between derivational priming and control, and between coordinate-compound constitute priming and control, respectively (Figure 4C). Derivational structures (derivation minus control, 0.279 ± 0.350 µV) elicited a greater P250 than compound structures (−0.354 ± 0.250 µV) (*p*s < 0.05) in both hemispheres. Interestingly, P250 in the right hemisphere (0.206 ± 0.294 µV) was statistically higher than that from the left hemisphere (−0.280 ± 0.261 µV) (*p* < 0.05). The P250 patterns in the midline were consistent across bilateral sites. With regard to the N400 component, the patterns remained unchanged.

### 3.3. fNIRS Results

**Figure 5:**
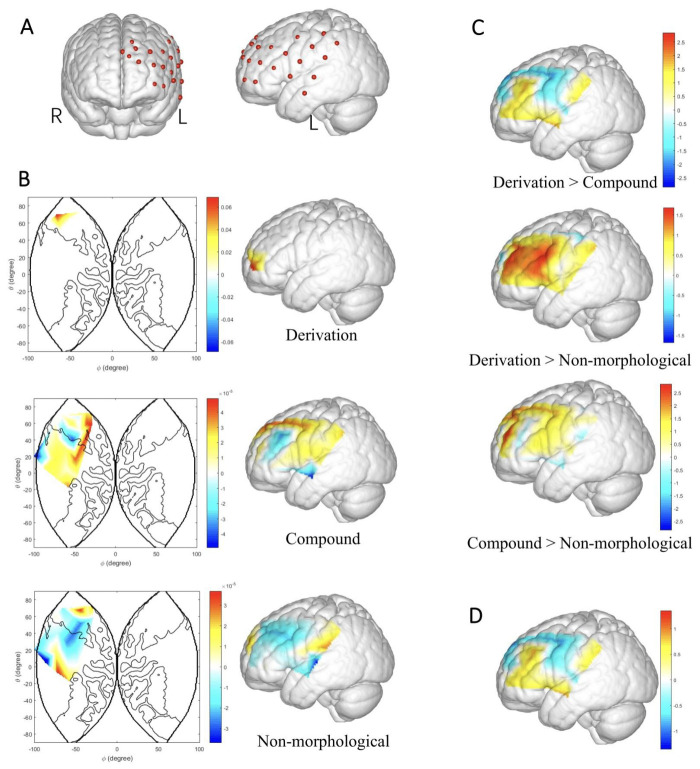
Configuration of fNIRS layout and activation maps. (**A**) fNIRS layout covering the frontal and temporal cortex of the left hemisphere, from the frontal and the left views, respectively. (**B**) Brain activation patterns in reading words across three conditions based on HbO beta values. (**C**) T maps of three pairwise comparisons. (**D**) Activation difference of pure word structure effect between Derivation vs. Control, and Compound vs. Control.

Although both HbO and HbR beta values were generated from the GLM estimations, the present study only analyzed the HbO signals to examine the lexical condition effect [68,69]. Firstly, HbO beta values were compared across the three conditions by repeated-measures ANOVA channel by channel, among which channel 6 in the frontopolar area showed a significant condition effect, *F*(2, 56) = 3.162, *p* < 0.05, partial *η*^2^ = 0.101. Pairwise comparisons were then conducted across the three conditions. Table 2 summarized significant (*p* < 0.05) results when *p* values were not corrected. Specifically, the left frontal cortex, including the dorsolateral prefrontal cortex (DLPFC), frontopolar area, and the frontal eye fields (channels 5, 6, and 11), exhibited greater activation from non-morphological priming to compound constitute priming. The associated brain activation patterns were visualized in Figure 5B,C.

Furthermore, pure word structure effect (derivation/compound minus non-morphological) was also examined, and the *t* tests results were plotted in Figure 5D. The comparison obtained no significant results.

### 3.4. Correlational Results

**Figure 6:**
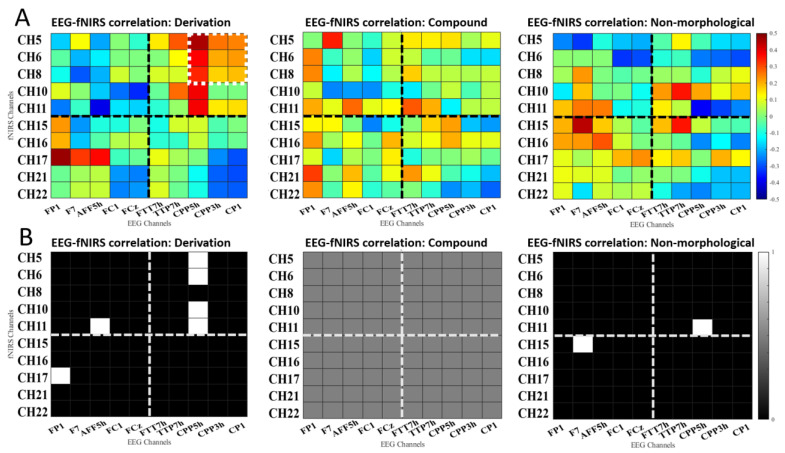

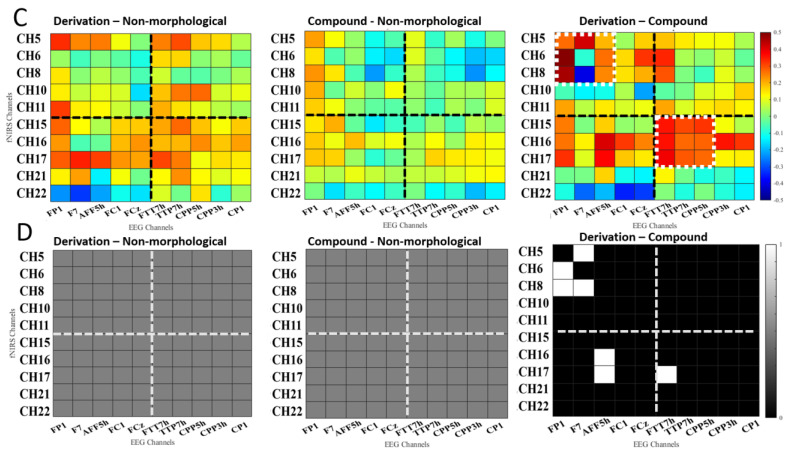
Correlation coefficient matrixes between electrophysiological responses (ERP data) and brain activation (fNIRS data). Dashed line represents the division of frontal (ERP: FP1, F7, AFF5h, FC1, FCz; fNIRS: CH5, CH6, CH8, CH10, CH11) and temporal regions (ERP: FTT7h, TTP7h, CPP5h, CPP3h, CP1; fNIRS: CH15, CH16, CH17, CH21, CH22). (**A**) Correlations between ERP and fNIRS data among representative probes across the three conditions. Brighter color stands for stronger correlation. The white square with dashed outline represents region of interest. (**B**) Binary maps of the correlation results across the three conditions. The white squares denote a significant correlation between bi-modal data (*p* < 0.05). (**C**) Correlations between ERP and fNIRS data among representative probes across the three comparisons. (**D**) Binary maps of the correlation results across the three comparisons (*p* < 0.05).

To examine the alignment of ERP and fNIRS data over the left fronto-temporal cortex, the relationships between P250 and HbO beta weights were examined by calculating the Pearson correlation coefficients. Specifically, mean ERP amplitudes of FP1, F7, AFF5h, FC1, FCz, and fNIRS hemodynamic responses on channels 5, 6, 8, 10, and 11 were used to depict brain activation in the left frontal cortex, respectively, while the brain activities in the left temporal region were accessed from EEG channels (FTT7h, TTP7h, CPP5h, CPP3h, CP1) and fNIRS channels (CH15, CH16, CH17, CH21, CH22). According to the matrixes of correlation coefficients, P250 well predicted the temporal activation in derivational priming (Figure 6A,B), which implicates a broad semantic network. More importantly, both spatial (frontal) and temporal ROIs (Figure 6C,D) showed close associations between P250 and hemodynamic responses in derivation vs. compound contrast in the left fronto-temporal region.

## 4. Discussion

The current study examined the temporo-spatial brain activation patterns associated with the Chinese word structure effect (derivation vs. compound vs. non-morphological) by using a masked priming technique in a lexical decision task. The analysis of behavioral performance revealed morphological priming facilitation for coordinate-compounds, which exhibited statistically higher accuracy and a relatively shorter reaction time than non-morphological pairs. This pattern extended the findings from Chung et al. [3] by suggesting that the coordinate structure priming effect could not only be elicited from word pairs sharing the same structure but also from coordinate words that are primed by their first roots, conditional on a short SOA (57 ms). Nevertheless, this morphological facilitation was not in line with Liu and McBride-Chang [45] and Cui et al. [50]. The discrepancy could be attributed to the relatively longer SOA and different task demands, such that Liu and McBride-Chang [45] used 200 ms for SOA and Cui et al. [50] asked participants to respond to both primes and targets in a self-paced manner. The current findings therefore implicate that the Chinese coordinate structure effect might be sensitive to SOAs and index an automatic and short-lived activation of morphological information at an early stage of lexical access [45]. Yet, the present study failed to detect any morphological priming effect for Chinese derivations. As there was no previous study examining the psychological reality of Chinese derivations and the morphological structure effect was somehow weak in behavioral patterns due to technical limitations [3,50], we will rely more on ERP and imaging data to advance understandings of this word structure.

Specifically, both reaction time and accuracy data revealed that derivations might be harder to recognize than coordinate-compound structures in constitute priming scenarios. This finding is somewhat consistent with the coordinate vs. subordinate structure contrast identified in the semantic priming paradigm [45]. Both subordinate structure (e.g., 黑板, black-board, “blackboard”) and derivation (e.g., 作家, writing-expert, “writer”) employed a modifier-head relation, whereas the suffix in derivation (i.e., 家 in 作家) has been delexicalized in grammaticalization process. As a result, the suffix is loosely attached to the base form and productive in deriving new word forms (e.g., 画家, “painter”; 艺术家, “artist”; 音乐家, “musician”). In contrast, the inter-connection between the constituents within a coordinate structure is relatively stronger than the base-suffix association in a derivation, though not as strong as those of subordinate compounds [46]. Therefore, the word structure effect identified in the current behavioral results sheds light on the spreading activation account, where the strengths between constituents within a morphologically complex word would determine the cognitive efforts needed in word recognition.

The ERP data revealed a prominent word structure effect on the frontal P250. Difference wave analyses (derivation/coordinate-compound constitute priming minus non-morphological relationship) showed that derivations elicited significantly greater bilateral positivities in the time window of 220–300 ms, compared with coordinate-compounds, while the right hemisphere tends to be more significant. The frontal P250 effect was previously found in the comparison between word pairs of different semantic relatedness [51] and structural consistency [3]. Importantly, this early component was sensitive to SOA, as it was only present in a short SOA of 150 ms/57 ms, yet not in the 700-ms condition, which indexes the automatic access in lexical processing. Existing MEG studies [71] also associated this early time window with a decomposition and parsing operation in morphological processing. As such, relative to coordinate-compounds, greater P250 amplitudes found in derivations of the current study would indicate more cognitive resource consumption in morphological parsing, which confirmed the behavioral patterns. By virtue of the spreading activation account, all concepts (i.e., word roots in the current case) are stored as nodes and connected with each other by different weights. With the input of prime, not only the corresponding morphemes but also the candidate morpheme, with which it could make up new words, would be activated, followed by the re-combination of two morphemes. Yet, the inter-constitute connection across word structures is different. For instance, compared with subordinate structures, both constitutes in the coordinate structure contribute equally to the whole word, manifesting a rather loose connection [46]. The current P250 effects further suggested that Chinese derivations and compounds could be discriminated in the frontal cortex in light of their differing inter-constitute relationships, as facilitated by spreading activation.

In addition to P250 effects, Chung et al. [3] also reported a classic N400 semantic priming effect mediated by semantic relatedness between primes and targets, which was absent in the current study. According to the grand-average brainwaves and topographies, there was a N400-like component, which was widely distributed in the centro-parietal cortex. Yet, the comparison between three conditions did not reach any statistical difference. It might be due to the fact that the current study did not manipulate the semantics, as the semantic associations between primes and targets were generally comparable by measuring and matching semantic transparency. The frontal P250 effect could therefore be attributed to a relatively purer word structure modulation.

Brain activation obtained from fNIRS data provided robust evidence on morphological priming effect in compound word recognition, which was not statistically reliable in reaction time. Coordinate-compounds elicited enhanced activation than the controls in the left frontal network, including the DLPFC and frontopolar area. This pattern identified in the left frontal cortex from the current study is roughly consistent with previous fMRI results associated with morphological processing for children and/or with auditory stimuli. For instance, word pairs sharing morphemic information (e.g., 高温-高空, high temperature-high sky) showed the highest activation in the left inferior frontal gyrus (IFG) across all lexical conditions in an explicit auditory morphological judgment task [36]. This implicates an important role for the left IFG in morphological processing across alphabetic languages [72,73] and Chinese. In a recent bilingual children study [52], Chinese-English bilingual children elicited greater activation in the IFG in an auditory English morphological task (e.g., re-jump), compared with a Chinese compound morphology task (e.g., 病花, sick-flower). This pattern was similar in monolingual English children, further highlighting the role of the left frontal cortex in morphological processing. Compared with fMRI, fNIRS is relatively limited in spatial resolution, which might explain the current study’s failure to localize subtle changes in the exact IFG. Yet, the left frontal cortex (mostly DLPFC) still showed manifest activations in morphological priming conditions. Importantly, the current findings could justify previous fMRI results with data from print among the adult population. Taken together, we may infer that the left frontal cortex implicates a core area for morphological processing across language modalities and literacy stages.

Whilst this study did not include as many word structures, it did partially substantiate a word structure effect between Chinese derivations and coordinate-compounds, which made an original contribution to the field. In particular, correlational analysis on derivation vs. compound difference revealed a good degree of coherence between frontal P250 values and hemodynamic responses in the left frontal area. Derivations generated stronger activation than compounds, given their rather loose connections between roots and suffixes, which might demand more efforts in constitute meaning access and re-composition. The fNIRS data is overall consistent with ERP results, which further validates the word structure effect in the frontal cortex.

Nevertheless, unlike previous MEG and fMRI studies [2,53], the current study did not detect exact temporal engagement in word structure differentiation under masked priming settings. Yet, ERP-fNIRS correlations showed that P250 in the temporal cortex could well predict brain activations in the corresponding regions. Meanwhile, the electrophysiological responses in the left temporal region were closely associated with frontal activations. While we highlighted the role of the left frontal cortex in word structure discrimination, the temporal areas should not be neglected. Future studies could address this region by using fMRI techniques and functional connectivity analysis.

The current findings do not apply to the dual-route theory in morphologically rich languages [8,16]. According to this theory, the recognition of regular inflections and rule-based word units requires an online computation mechanism and employs the left fronto-temporal cortex. In contrast, storage-based and highly lexicalized items engage broader bilateral brain regions, which are less time-consuming. However, Chinese is a language lacking morphological inflection. Instead, compounding is the dominant structure of Chinese morphology and implicates a storage-based representation. Even though the proportion of derivations is much smaller than that of compounds, they are highly productive given the adhesiveness of affixes. By comparing two-word structures with differing lexicalization extents, the current study found that Chinese derivations might employ broader brain networks in bilateral fronto-temporal cortex, while compounds were mostly manifested in the left frontal regions and indexed by a storage-based mechanism. This brain pattern might be attributable to the connection strength between constituents across different word structures in light of spreading activation theory.

In conclusion, this study examined the Chinese word structure effect in a masked priming paradigm by using EEG-fNINS simultaneous data. Specifically, we found prominent word structure effects in the frontal cortex. Derivations elicited significantly greater bilateral positivities (i.e., P250) in the time window of 220–300 ms than coordinate compounds, while the right hemisphere tends to be more significant. Overall, Chinese derivations exhibited significantly enhanced brain activation in the frontal cortex and involved broader brain networks as compared with lexicalized compounds. As a language impoverished of grammatical morphology, Chinese word structure effect showed a distinct pattern from the dual-route mechanism in alphabetic languages. Reading scientists might need to take the idiosyncrasy of Chinese morphology into account when concluding on the universality of morphological processing across languages.

Finally, a number of limitations should be considered. First, we only included coordinate compounds and derivations to examine the word structure effect. It would be interesting to examine whether this pattern would extend to other sub-types of compounding (e.g., verb-object, verb-resultative) and reduplication structures. In addition, even though fNIRS manifests good ecological validity and compatibility with EEG, its spatial resolution is lower than that of MEG and fMRI. Future research needs to be done to examine our findings by including more morphological structures and using more nuanced imaging techniques.

## Figures and Tables

**Table 1:** Stimulus statistics across the three lexical conditions (standard deviation in parentheses).

	Prime	Frequency	Stroke Number	Target	Frequency	Stroke Number	Cloze Probability	Semantic Relatedness
Derivational word	网/wang3/, net	94 (145)	8.3 (2.3)	网民/wang3 min2/, netizen	8 (32)	15.3 (3.8)	0.07 (0.12)	5.2 (0.9)
Compound word	花/hua1/, flower	83 (109)	8.6 (3.1)	花草/hua2 cao3/, plant	7 (10)	16.9 (4.6)	0.12 (0.19)	5.4 (0.9)
Non-morphological	脏/zang1/, dirty	356 (1076)	8.8 (2.8)	污水/wu1 shui3/, dirty water	34 (79)	17.8 (4.5)	/	5.3 (0.5)

**Table 2:** Comparison results of significant channels and their corresponding spatial information.

CH#	MNI Coordinates			BA	Anatomical Label	Overlap	Comparisons	*t*	*p* (Uncorrected)	*p* (FDR Corrected)
	x	y	z							
5	−21	67	53	9	Dorsolateral prefrontal cortex	0.8178	Compound > Non-morphological	2.42	0.02	0.07
6	−44	67	27	10	Frontopolar area	0.95455	Compound > Non-morphological	2.84	0.01	0.02
11	−15	61	61	8	Includes frontal eye fields	0.77686	Compound > Non-morphological	2.07	0.05	0.14

CH#: channel number, BA: Brodmann area, FDR: false discovery rate [70].

## Data Availability

Data is available on request.
